# Commencement Exercises

**Published:** 1854-03

**Authors:** 


*Commencement Exercises.*—The annual commencement of the Medical Department of the University of Buffalo, was held at the American Hall, on Wednesday evening, February 22d.

The attendance was large, the hall being nearly filled witn the elite of Buffalo, although the night was very inclement. Probably hardly a hundred more could have been seated.

Prof. Jas. P. White, Dean of the Faculty, announced the order of exercises.

The Rev. Dr. John C. Lord then opened with a most impressive prayer, imploring the blessing of the Almighty upon the institution, its teachers, and alumni.

The Hon. Millard Fillmore, Chancellor of the University, then addressed the class in a tone of earnest advice, as to honesty and probity of purpose, in their future career; urging upon them the importance and dignity of the profession of their choice, and reminding them that it was no longer fenced in by restrictive laws—that it had no governmental privileges—and must rely upon its own worth and usefulness to make itself appreciated. A diploma conferred no rights which could not be obtained without it. It was a simple letter of recommendation, to be credited only as the conduct of its bearer vindicated its truth.

The presence of the distinguished chancellor, who has so long been separated from collegiate and Buffalo interests, by his tenure of the highest office in the gift of the nation, added grace and interest to the proceedings. The graduates received their degrees from one whose own life is a fit example to be followed by whoever would wish to rise through merit, industry, and probity of purpose.

The chancellor then conferred, in due form, the degree of Doctor of Medicine upon twenty-four young gentlemen, as follows:

Jerome Punderson Avery, Auburn, Cayuga Co., N. Y.;

Subject of Thesis—Typhoid Fever.

Perry Barrett, Kingsville, Ashtabula Co., Ohio.;

Influence of Mind over the Body and the Action of Medicines.

John Howard Cole, Wells, Bradford Co., Penn.;

Bilious Remittent Fevers.

William Henry Cruikshank, Ogdensburgh, St. Lawrence Co., N. Y.

Consumption.

Horatio Nelson Caner, Warsaw, Wyoming Co., N. Y.;
Hæmorrhage in the Nervous Centers.
John G. Collver, Simcoe, Canada West;
The Medical Profession.
George Coatsworth, County of Kent, Canada West;
History of Human Life.
Edward Hatch Davis, Parma, Monroe Co., N. Y.;
Reflections of a Candidate for Graduation.
Waldow Curtiss Daniels, Buffalo, N. Y.;
Recent Sprains.
Franklin Goodyear, Cortlandville, Cortland Co., N. Y.;
Dysentery.
Fred. Gardner, Buffalo N. Y.;
Acute Rheumatism.
John Wicks Heywood, Buffalo, N. Y.;
——— ——— ———
David W. Hershey, Williamsville, Erie Co., N. Y.;
Omne Vivum ex Ovo.
Robert James Johnston, Thorold, Canada West;
——— ——— ———
Restcome Rogers Kirby, Monroe, Michigan;
Miasmatic Fever.
Jonas Wellman Lyman, Waterville, Lycoming Co., Pa.;
The Genuine Physician.
Duncan McLochlin, Caledonia, Livingston Co., N. Y.;
The Obstetrician.
Dugald McKellar, Wardsville, Canada West;
Headache.
William Andrew Newell, Buffalo, N. Y.;
Abortion.
Luman Pettibone Lawrence Parker, Clarence, Erie Co., N. Y.;
Typhoid Fever.
Joseph Warren Sawyer, Freedom, Cattaraugus Co., N. Y.;
Puerperal Peritonitis.
Clark Smith, Hartford, Washington Co., N. Y.;
Scrofula and Scrofulous Ophthalmia.
Joel Underhill, Buffalo, N. Y.;
Hypertrophy of the Heart.
Joshua Whitney, Eagle Village, Wyoming Co., N. Y.;
Searlatina.

The Honorary Degree of Doctor of Medicine was also conferred upon the Hon. Daniel P. Bissell, of Utica.

The address to the graduates, by Prof. Thomas F. Rochester, we need not dwell upon, as we hope to present it, *in extenso*, to our readers, in our next number, the graduating class having requested its publication. We need only say here, that it was listened to with the deepest interest by the entire audience.

A benediction, from the Rev. Montgomery Schuyler, closed the exercises, which had been interspersed with choice music under the direction of Albert Poppenberg. H.

